# Facial Growth Patterns and Midpalatal Suture Maturation in Adults: A Retrospective CBCT‐Based Observational Study

**DOI:** 10.1111/ocr.70118

**Published:** 2026-03-27

**Authors:** Elizabety do Nascimento Silva, Nathalie Murielly Rolim de Abreu, Frederico Barbosa de Sousa, Francine Kuhl Panzarella, Rudyard dos Santos Oliveira

**Affiliations:** ^1^ Dentistry Department Federal University of Paraíba Paraíba Brazil; ^2^ Postgraduate Program in Dentistry Federal University of Paraíba Paraíba Brazil; ^3^ Morphology Department Federal University of Paraíba Paraíba Brazil; ^4^ Oral Radiology Division Faculdade São Leopoldo Mandic Campinas SP Brazil

**Keywords:** bone maturation, cone‐beam computed tomography, facial growth, midpalatal suture, miniscrew‐assisted maxillary expansion

## Abstract

**Objective:**

To investigate whether facial growth pattern is associated with the maturation stage of the midpalatal suture (MPS) in adults using cone‐beam computed tomography (CBCT).

**Materials and Methods:**

This retrospective study reviewed 300 full‐skull CBCT scans acquired between January 2018 and May 2023, of which 120 met the eligibility criteria. Individuals aged 18 years or older were classified according to facial growth pattern (brachyfacial, mesofacial or dolichofacial) using Ricketts' VERT index. Age was categorised into < 30 and ≥ 30 years based on clinical evidence suggesting increased frequency of sutural fusion after the third decade of life. Exclusion criteria included craniofacial anomalies, syndromic conditions, systemic or hormonal disorders affecting skeletal growth, impacted teeth, previous maxillary surgery, trauma and inadequate image quality. MPS maturation was assessed using Angelieri's five‐stage classification (A–E). Two calibrated examiners independently evaluated all cephalometric and tomographic records. Ordinal multinomial logistic regression was applied to analyse associations between facial growth pattern, age, sex and MPS maturation stage.

**Results:**

Facial growth pattern was significantly associated with MPS maturation stage (χ^2^ = 20.65, *p* < 0.001), with dolichofacial individuals more frequently presenting earlier maturation stages (B or C). Age (*p* = 0.297) and sex (*p* = 0.870) were not significantly associated with MPS maturation in this adult sample. Reliability analysis demonstrated excellent intra‐ and inter‐examiner agreement (ICC = 0.95; κ = 0.98).

**Conclusion:**

Facial growth pattern was associated with MPS maturation stage in adults and may function as an adjunctive diagnostic parameter. Dolichofacial individuals more frequently exhibited earlier stages of sutural maturation, whereas age and sex showed no significant association. Although facial pattern analysis should not replace CBCT evaluation, it may contribute to a more individualised, anatomy‐based diagnostic assessment. These findings should be interpreted cautiously due to the cross‐sectional design and specific population studied.

## 1 Introduction

The midpalatal suture (MPS) is a central component of the maxillary complex, playing a fundamental role in transverse maxillary development and craniofacial growth. During childhood and adolescence, the MPS remains relatively patent, allowing skeletal adaptation in response to orthopaedic forces. As skeletal maturation progresses, the suture undergoes a gradual biological process of interdigitation and ossification, which may culminate in partial or complete fusion. Importantly, this process does not occur uniformly across individuals and is influenced by multiple factors beyond chronological age, including genetic background, craniofacial morphology, functional loading and systemic growth patterns [1, 2, 3].

A critical distinction must be made between individuals who are still undergoing active craniofacial growth and those who have reached skeletal maturity. In growing patients, sutural patency is expected, and the MPS behaves as a growth site that responds predictably to orthopaedic stimuli. In contrast, adults present a biologically different condition, in which the suture represents a remodelling interface rather than a growth centre. In this context, the clinical challenge is not whether growth can be stimulated, but rather how advanced the ossification process is and how much resistance the suture and surrounding structures may offer. Failure to clearly differentiate these biological phases may lead to misinterpretation of maturation stages and inappropriate extrapolation of findings from adolescents to adults [1, 4, 5, 6, 7].

Cone‐beam computed tomography (CBCT) has enabled direct visualisation of the MPS and led to the development of imaging‐based diagnostic classifications. Among these, the five‐stage maturation system proposed by Angelieri et al. has been widely adopted to describe morphological changes ranging from a clearly visible, non‐interdigitated suture (stages A and B) to partial (stage C) and advanced or complete fusion (stages D and E). While this classification provides a practical diagnostic framework, growing evidence suggests that its interpretation should not be restricted to the MPS alone, as circummaxillary sutures and overall craniofacial morphology also contribute to skeletal resistance and maturation patterns [8, 9, 10, 11].

Chronological age has traditionally been considered a proxy for sutural maturation; however, several studies have demonstrated substantial interindividual variability, particularly in adults. Individuals of similar age may present markedly different maturation stages, indicating that age alone is an unreliable predictor of MPS ossification. Sex‐related differences have also been investigated, with some studies suggesting earlier maturation in females, possibly related to smaller maxillary dimensions and earlier skeletal development, although results remain inconsistent [9, 10, 11, 12, 13, 14].

In recent years, attention has shifted towards craniofacial morphology as a potential determinant of sutural maturation. Facial growth pattern reflects the vertical and horizontal balance of the craniofacial skeleton and is associated with differences in bone density, muscular function and biomechanical loading. Previous studies and clinical experience suggest that individuals with a dolichofacial pattern tend to exhibit delayed skeletal maturation when compared with brachyfacial counterparts. Although this concept is not new, its specific relationship with MPS maturation in adults remains insufficiently explored, particularly from a strictly diagnostic perspective [15, 16, 17, 18, 19, 20].

Most available studies addressing MPS maturation have focused on treatment outcomes or expansion techniques, rather than on identifying reliable diagnostic indicators that may assist in predicting sutural status before intervention. From a diagnostic standpoint, understanding whether facial growth pattern is associated with MPS maturation could provide a low‐cost, radiation‐free adjunct to CBCT interpretation, helping clinicians contextualise tomographic findings within the broader framework of craniofacial biology [21, 22, 23, 24, 25, 26].

Therefore, the objective of the present study was to investigate the association between facial growth pattern and MPS maturation stage in adults using CBCT imaging, and to determine whether facial morphology may serve as a predictive indicator of sutural status. The null hypothesis tested was that the facial growth pattern is not associated with the maturation stage of the MPS in adult individuals.

## 2 Materials and Methods

### 2.1 Sample Determination

Three hundred CBCT scans of the entire skull were retrospectively reviewed from January 2018 to May 2023. All scans had been originally obtained for diagnostic purposes related to orthodontic or orthognathic evaluation. The use of full‐skull CBCT was justified by clinical indication and institutional protocols and allowed the simultaneous assessment of cephalometric parameters and MPS morphology.

From the initial pool of 300 scans, 168 were excluded for not meeting the eligibility criteria. Exclusions were due to poor image quality, incomplete permanent dentition, craniofacial anomalies, history of trauma, previous maxillary or orthognathic surgery, presence of lesions in the region of interest, impacted teeth, significant dental or skeletal asymmetries that could interfere with cephalometric analysis or sutural evaluation, and systemic or hormonal conditions known to influence skeletal growth or bone metabolism.

After application of the inclusion and exclusion criteria, 132 CBCT scans met all eligibility requirements. From these, 120 scans were selected through stratified allocation to ensure equal representation across facial growth patterns.

The final sample consisted of 120 adult individuals selected by stratified allocation into three equal groups (*n* = 40 each), according to facial growth pattern: brachyfacial, mesofacial and dolichofacial. This stratification strategy ensured balanced distribution of skeletal patterns rather than random selection from the eligible cases.

Age and sex were not used as stratification variables because their distribution among the 132 eligible scans was homogeneous across facial growth patterns. Additional stratification by these variables would have resulted in unequal group sizes and reduced statistical power. Instead, age and sex were included as covariates in the statistical analysis.

Participants were further categorised into two age subgroups (< 30 years and ≥ 30 years). This cutoff reflects longstanding clinical observations that MPS fusion becomes more frequent after the third decade of life, a finding supported by previous studies demonstrating progressive ossification and reduced sutural patency in adult populations [17, 18].

The final sample size provided a statistical power of 0.80 (β = 0.20) at a significance level of 0.05 (α = 0.05) to detect medium effect sizes (w = 0.5) in multinomial logistic regression analyses involving sex, age group, facial growth pattern and MPS maturation stage. Power calculation was performed using Jamovi software (version 2.4.14, Sydney, Australia). Although conducted retrospectively, this analysis confirmed the adequacy of the selected sample size for the study design.

All CBCT images were acquired using a standardised imaging protocol with an i‐CAT scanner (Imaging Sciences International, Hatfield, PA), operating at 12‐bit grey‐scale resolution, with a voxel size of 0.25 mm and a field of view (FOV) of 13 × 22 cm. Images were obtained under standardised conditions, with the head positioned according to the Frankfort horizontal plane.

To standardise tomographic assessment, all images were independently analysed by two previously calibrated examiners. Calibration was performed in two sessions separated by a 2‐week interval and included both cephalometric tracing and classification of facial growth pattern and MPS maturation stage. Reliability was assessed separately for facial growth pattern classification and for the Angelieri MPS staging protocol using intraclass correlation coefficients (ICC) and Cohen's kappa statistics (Table 1).

**TABLE 1:** Intraobserver and interobserver reliability analysis for facial growth pattern classification and Angelieri midpalatal suture (MPS) maturation staging. Intraclass correlation coefficients (ICC) were calculated using a two‐way mixed‐effects model with absolute agreement. Cohen's kappa statistics were used for categorical agreement analysis of MPS stages. Agreement was interpreted according to conventional thresholds (ICC > 0.90 = excellent; κ > 0.81 = near‐perfect).

Variable evaluated	Reliability type	Examiner	(*) ICC (95% CI)	(*) Cohen's κ	*p*	Interpretation
Facial Growth Pattern	Intraobserver	Examiner 1	0.94 (0.90–0.97)	—	< 0.0001	Excellent
Facial Growth Pattern	Intraobserver	Examiner 2	0.96 (0.92–0.98)	—	< 0.0001	Excellent
Facial Growth Pattern	Interobserver	—	0.95 (0.91–0.98)	—	< 0.0001	Excellent
Angelieri MPS Staging	Intraobserver	Examiner 1	0.95 (0.92–0.98)	0.97	< 0.0001	Excellent/Near‐perfect
Angelieri MPS Staging	Intraobserver	Examiner 2	0.96 (0.93–0.98)	0.98	< 0.0001	Excellent/Near‐perfect
Angelieri MPS Staging	Interobserver	—	0.95 (0.92–0.98)	0.98	< 0.0001	Excellent/Near‐perfect

### 2.2 Determining Facial Growth Patterns

Facial growth pattern was determined using the VERT index proposed by Ricketts, following the original cephalometric protocol, a composite cephalometric indicator designed to characterise vertical skeletal morphology. The VERT index is calculated from five cephalometric measurements: facial axis, facial depth, mandibular plane angle, lower facial height and mandibular arch. Together, these variables generate a numerical value that reflects the individual's vertical growth tendency and skeletal balance.

Based on the composite VERT index value, individuals were classified into three facial growth patterns: brachyfacial (VERT > + 0.5), mesofacial (VERT between −0.5 and + 0.5) and dolichofacial (VERT < −0.5). These thresholds are consistent with the original description of the VERT index and have been widely applied in orthodontic and craniofacial research.

Lateral cephalometric images used for facial growth assessment were reconstructed from CBCT datasets in DICOM format using Dolphin Imaging software (version 11.0; Dolphin Imaging Systems, USA). Two‐dimensional lateral cephalograms were generated by aligning the midsagittal plane and standardising head orientation according to the Frankfort horizontal plane, using standardised projection parameters to ensure reproducibility across reconstructions.

Following image reconstruction, the cephalometric landmarks required for VERT index calculation were manually identified by the calibrated examiners in accordance with Ricketts' protocol. The software provided guided landmark identification, sequential point display and regional magnification to improve marking accuracy and minimise the effects of anatomical superimposition. Once all landmarks were defined, the software automatically generated the cephalometric tracing and calculated the corresponding linear and angular measurements (Supporting Information).

The final composite VERT index value obtained for each participant was used to classify facial growth patterns and served as the categorical variable for subsequent statistical analyses.

### 2.3 Analysis of Maturation Stages

All CBCT images were processed and evaluated using OnDemand3D software (CyberMed, Seoul, Republic of Korea). Assessment of MPS maturation followed the five‐stage classification system originally proposed by Angelieri et al. [18], which describes progressive morphological changes in sutural appearance from complete patency to advanced ossification and has been widely applied in previous imaging‐based studies.

MPS maturation was evaluated in the central transverse axial dimension of the palate, with inspection performed in the superior–inferior direction. For each individual, a standardised midsagittal cross‐sectional slice was selected by identifying the plane passing through the midline of the palate and orienting it perpendicular to both axial and coronal planes. This orientation allowed consistent visualisation of the MPS along its entire anteroposterior extent.

To minimise observer bias and maintain examiner blinding, image selection and export were performed by an independent researcher who was not involved in the classification process. The selected axial cross‐sections were anonymised, coded and organised into a PowerPoint 365 presentation with a black background. Images were displayed sequentially on a high‐definition monitor in a controlled darkened environment to optimise visual assessment conditions. The order of image presentation was randomised independently for each observer.

For each patient, two axial slices were analysed: a central reference slice and an auxiliary slice to aid interpretation of palatal curvature, suture thickness or transitional morphology. No adjustments were made to image brightness or contrast. Additionally, each image was provided in two formats—original and with a negative filter applied—to enhance visual discrimination of sutural boundaries and adjacent bone structures.

To further reduce subjective variability and to support the ordinal interpretation of maturation stages, an auxiliary grey‐scale analysis of the MPS was performed using ImageJ software (National Institutes of Health, Bethesda, MD). A standardised region of interest (ROI) was positioned along the anterior two‐thirds of the suture in axial slices where sutural margins were clearly identifiable. Mean grey‐scale values were extracted as an indirect indicator of relative mineral density, with higher values corresponding to increased radiopacity and more advanced ossification. These quantitative data were not included as predictors in the regression models but served as an internal reference to verify consistency between visual stages and to reinforce the reliability of the morphological classification.

**FIGURE 1:**
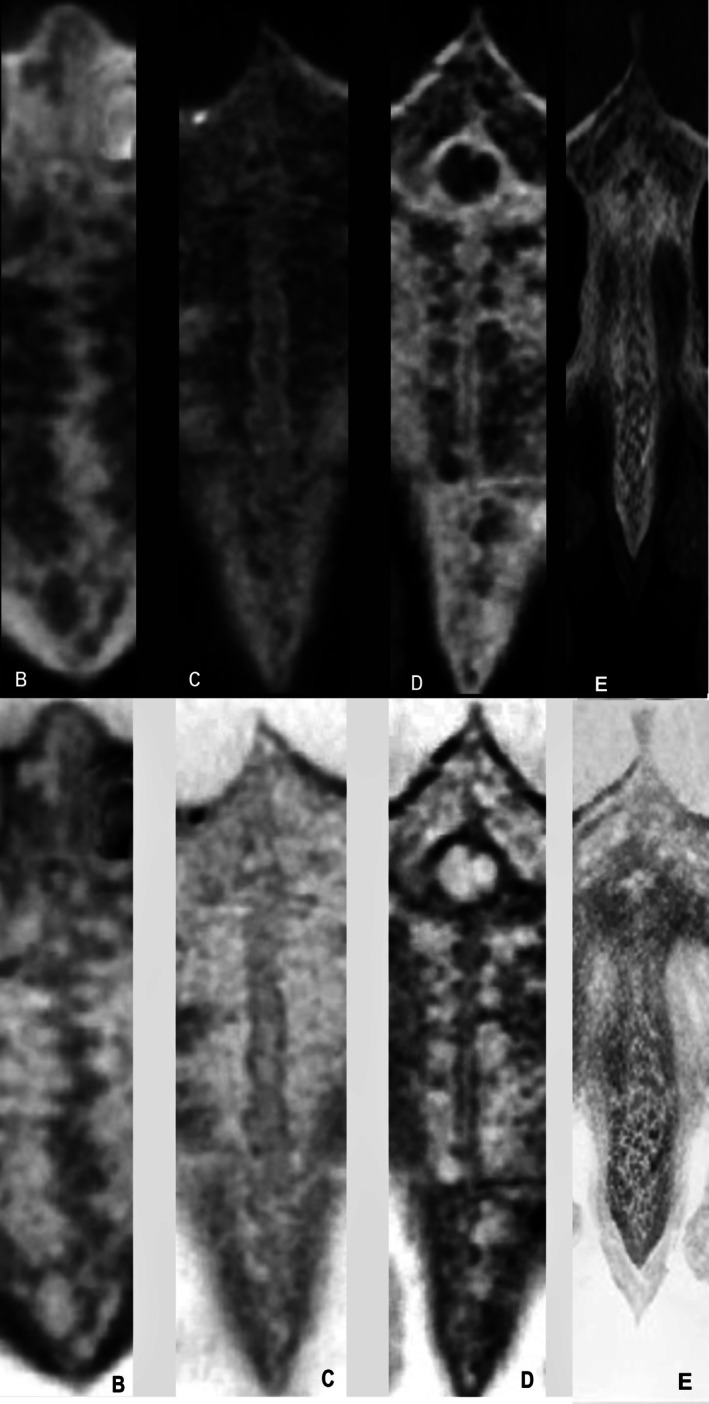
Representative cone‐beam computed tomography (CBCT) images of midpalatal suture maturation stages (B, C, D and E). Top row: Unfiltered images; bottom row: Images displayed with a negative filter to enhance suture boundary visualisation.

All images were independently and blindly scored by two previously calibrated examiners according to the five maturation stages (A–E) described by Angelieri et al. [11]. Examiners had no access to patient demographic, clinical or cephalometric information during the evaluation process. All scores were recorded independently, and disagreements were resolved only after completion of the reliability analysis (Figure 1).

### 2.4 Statistical Analysis

Statistical analyses were performed using Jamovi software (version 2.4.14; Sydney, Australia). An ordinal multinomial logistic regression model was applied to investigate the association between selected explanatory variables and the maturation stage of the MPS. The dependent variable consisted of the five ordered maturation stages (A–E) described by Angelieri, while sex, age group and facial growth pattern were included as independent variables.

The regression model estimated the effect of each predictor on the probability of an individual being classified into progressively advanced MPS maturation stages. This modelling approach is appropriate for ordered categorical outcomes, as it accounts for the inherent ranking of the response variable while allowing simultaneous evaluation of multiple covariates and their relative contributions.

Age was entered as a categorical variable (< 30 years and ≥ 30 years), based on previous evidence indicating a higher frequency of MPS fusion after the third decade of life [17, 18]. Although age could theoretically be treated as a continuous variable, the categorical approach was selected to enhance clinical interpretability and to reflect commonly used thresholds in adult orthodontic assessment. A sensitivity analysis considering age as a continuous predictor was explored but not retained in the final model due to lack of statistical significance and because dichotomisation provided a more pragmatic clinical stratification within the adult sample.

Given the ordinal nature of the outcome variable, the model estimated threshold parameters (B|C, C|D and D|E), which represent transition points between adjacent maturation stages. These thresholds assume the presence of an underlying continuous latent construct related to progressive sutural maturation, consistent with the biological process of ossification.

The auxiliary grey‐scale values obtained from ImageJ analysis were used as an internal reference to support the ordinal structure of the maturation stages. Higher grey‐scale values corresponded to increased radiopacity and more advanced ossification patterns. Although these quantitative measures were not incorporated as predictors in the regression analysis, they reinforced the biological plausibility of a latent continuum underlying the categorical staging system and supported the interpretability of the estimated thresholds.

Model fit was assessed using the Cox & Snell pseudo‐R^2^ statistic. Although the resulting R^2^ value was modest, such findings are common in regression models addressing complex biological processes influenced by multiple unmeasured factors. Importantly, a low pseudo‐R^2^ does not invalidate statistically significant associations observed between predictors and maturation stage transitions.

Statistical significance was set at *p* < 0.05 for all analyses. Results were reported as odds ratios (ORs) with corresponding 95% confidence intervals, providing an estimate of the strength and direction of the associations between explanatory variables and MPS maturation stage.

## 3 Results

The final sample consisted of 120 adults (62 females and 58 males), equally distributed among the three facial growth patterns (*n* = 40 each). The mean ages were 29.8 ± 6.1 years for brachyfacial individuals, 30.4 ± 5.9 years for mesofacial individuals and 30.2 ± 6.4 years for dolichofacial individuals. Across the age subgroups, 58 participants were < 30 years old (mean 24.3 ± 3.2), and 62 were ≥ 30 years old (mean 36.7 ± 4.1). No statistically significant differences were observed in age or sex distribution among the facial pattern groups (Table 2).

**TABLE 2:** Demographic and clinical characteristics of the study sample (*n* = 120), stratified by sex, age group (< 30 years; ≥ 30 years) and facial growth pattern (brachyfacial, mesofacial and dolichofacial).

Variable	Brachyfacial (*n* = 40)	Mesofacial (*n* = 40)	Dolichofacial (*n* = 40)	Total (*n* = 120)
Sex				
Female, *n* (%)	23 (57.5%)	25 (62.5%)	28 (70.0%)	62 (51.7%)
Male, *n* (%)	17 (42.5%)	15 (37.5%)	12 (30.0%)	58 (48.3%)
Age (years)				
Mean ± SD	29.8 ± 6.1	30.4 ± 5.9	30.2 ± 6.4	30.1 ± 6.1
Age group				
< 30 years, *n* (%)	24 (60.0%)	26 (65.0%)	24 (60.0%)	58 (48.3%)
≥ 30 years, *n* (%)	16 (40.0%)	14 (35.0%)	16 (40.0%)	62 (51.7%)

The multinomial logistic regression model demonstrated a statistically significant overall fit, with a chi‐square value of 22.1 (df = 4; *p* < 0.001), indicating that at least one predictor contributed meaningfully to the explanation of MPS maturation stages. The Cox and Snell R^2^ value (0.0451) indicated that only a small proportion of the variance in maturation stages was accounted for by the model—an expected finding given the biological complexity and multifactorial determinants of sutural ossification.

Likelihood ratio tests identified facial growth pattern as a statistically significant predictor of MPS maturation (χ^2^ = 20.6519, *p* < 0.001). In contrast, chronological age (χ^2^ = 1.0888, *p* = 0.297) and sex (χ^2^ = 0.0268, *p* = 0.870) were not significantly associated with maturation stage. These results suggest that skeletal morphology exerts a greater influence on MPS development than demographic variables in adults, all of whom were aged 18 years or older and thus beyond the stage of active sutural growth (Table 3).

**TABLE 3:** Goodness‐of‐fit test for the multinomial logistic regression model predicting midpalatal suture (MPS) maturation stage. The model demonstrated statistical significance, indicating that at least one of the predictors (sex, age group and facial growth pattern) contributed meaningfully to explaining MPS maturation stage/Likelihood ratio tests for individual predictors of midpalatal suture (MPS) maturation stage. Facial growth pattern was statistically significant, while chronological age and sex were not.

Parameter	χ^2^	df	Value	*p*
Overall Model Fit	22.1	4	—	< 0.001
Deviance	—	—	203	—
AIC	—	—	217	—
BIC	—	—	237	—
Cox & Snell R^2^	—	—	0.0451	—

Predictor effects (omnibus likelihood ratio tests)				
Age	1.0888	—	—	0.297
Sex	0.0268	—	—	0.87
Facial Type	20.6519	—	—	< 0.001

*Note:* The dependent variable (MPS maturation stage) was analysed as an ordinal outcome with the following order: B | C | D | E.

Model coefficients (Table 4) showed that individuals with a dolichofacial pattern were significantly less likely to present in advanced ossification stages (D or E) compared with mesofacial individuals (*β* = −1.3430; 95% CI: −2.272 to −0.459; *p* = 0.004). No significant differences were observed between brachyfacial and mesofacial patterns (*p* = 0.120), indicating a weaker association in that comparison.

**TABLE 4:** Multinomial logistic regression coefficients and 95% confidence intervals for predictors of midpalatal suture (MPS) maturation stage. Dolichofacial individuals were significantly less likely than mesofacial individuals to present in advanced stages (D or E). No significant difference was found between brachyfacial and mesofacial groups.

Predictor	Estimate	Lower	Upper	SE	Z	p	Odds ratio	Lower	Upper
		95% Confidence Interval						95% Confidence Interval	
Age									
> 30–< 30	0.4514	−0.393	1.327	0.436	1.035	0.301	1.571	0.675	3.770
Sex									
M–F	−0.0717	−0.926	0.800	0.438	−0.164	0.870	0.931	0.396	2.226
Facial_Type									
Brachyfacial–Mesofacial	0.8708	−0.199	2.029	0.560	1.554	0.120	2.389	0.820	7.608
Dolichofacial–Mesofacial	−1.3430	−2.272	−0.459	0.460	−2.918	0.004	0.261	0.103	0.632

The estimated model thresholds (Table 5) confirmed predictable transitions between maturation categories (B|C, C|D and D|E), with all thresholds highly significant (*p* < 0.001), except for the D|E transition, which approached significance (*p* = 0.056). This borderline result reflects greater biological variability during late‐stage fusion among adults and may explain why age—when categorised as < 30 or ≥ 30 years—did not emerge as a significant predictor, despite the established trend of increased fusion after the third decade of life.

**TABLE 5:** Model threshold parameters for transitions between midpalatal suture (MPS) maturation stages. All thresholds were highly significant (*p* < 0.001), except for the transition between stages D and E, which approached significance (*p* = 0.056).

Threshold	Estimate	SE	Z	*p*	Odds ratio
B | C	−4.081	0.697	−5.86	< 0.001	0.0169
C | D	−2.275	0.453	−5.02	< 0.001	0.1028
D | E	−0.737	0.385	−1.91	0.056	0.4784

Overall, the findings demonstrate that facial growth pattern is an independent predictor of MPS maturation in adults and may serve as a clinically relevant parameter to complement CBCT‐based decision‐making in maxillary expansion planning.

## 4 Discussion

The present study demonstrates a statistically significant association between facial growth pattern and MPS maturation stage in adults, highlighting facial pattern as a clinically relevant diagnostic parameter in the assessment of transverse maxillary conditions. Rather than proposing a predictive model for treatment outcomes, these findings contribute to a more refined understanding of anatomical variability in sutural maturation and its relationship with craniofacial morphology.

Unlike many previous studies that emphasised chronological age or sex as primary determinants of MPS maturation, neither variable showed a statistically significant association in the present model. This finding is consistent with earlier reports demonstrating substantial interindividual variability in sutural ossification and the limited reliability of demographic factors as isolated diagnostic indicators [2, 5, 12]. In contrast, facial growth pattern emerged as a relevant anatomical variable, suggesting that vertical skeletal morphology may reflect underlying differences in craniofacial development and sutural behaviour.

Recent CBCT‐based evidence has further demonstrated that MPS maturation is accompanied by measurable morphological changes of the palate rather than representing an isolated radiographic finding. In this regard, Kroselj Zevnik and Primozic (2025) [26] reported that palatal height and surface area are significantly greater in earlier MPS maturation stages (B and C) and progressively decrease in more advanced stages (D and E), while palatal length remains relatively stable across stages. These findings reinforce the interpretation of MPS maturation as a continuous anatomical process closely linked to maxillary morphology. The present results are biologically consistent with this evidence, as dolichofacial individuals—who characteristically present distinct vertical skeletal patterns and palatal configurations—were more frequently associated with earlier stages of sutural maturation. Taken together, these data suggest that facial growth patterns may indirectly capture underlying palatal morphological characteristics associated with MPS maturation, supporting an integrated, anatomy‐based diagnostic approach [11, 12, 13, 14, 15, 16, 17, 18].

The observation that dolichofacial individuals were more frequently associated with earlier maturation stages (B and C) is consistent with prior evidence describing variations in bone density, palatal vault morphology and craniofacial biomechanics across facial types [13, 14]. Diagnostic imaging studies of the palate have similarly shown that maxillary morphology differs according to skeletal pattern, reinforcing the concept that sutural maturation reflects broader craniofacial architecture rather than a purely chronological phenomenon [18, 19, 20, 21, 22, 23, 24, 25, 26].

From a diagnostic standpoint, understanding the maturation stage of the MPS is clinically relevant regardless of the expansion modality. Evidence from conventional rapid maxillary expansion (RME) suggests that earlier maturation stages (A–C) are associated with greater skeletal responsiveness, whereas advanced stages (D and E) reflect increased resistance and reduced orthopaedic effects [1]. Therefore, assessment of sutural maturation should be interpreted within a broader diagnostic framework rather than as a standalone determinant of treatment selection.

It is important to emphasise, however, that the Angelieri classification is based on morphological interpretation of CBCT images and is inherently subject to observer‐dependent variability and image quality constraints. Although widely applied in clinical research, this method does not directly quantify histological ossification, nor does it account for regional differences in sutural interdigitation or bone density along the anteroposterior extent of the palate [5, 16]. Furthermore, transitional stages—particularly between D and E—may represent a biological continuum rather than discrete categories, which may partially limit their discriminatory precision in adult patients. These limitations should be considered when interpreting the maturation stage as a diagnostic indicator rather than a definitive measure of skeletal resistance.

Other anatomical factors known to influence maxillary transverse resistance—such as palatal cortical thickness, circummaxillary sutural complexity and muscular dynamics—have been shown to vary with facial growth pattern [13, 21]. These structural differences support the interpretation that facial type reflects a constellation of morphological characteristics that may influence sutural maturation and craniofacial biomechanics, without implying a deterministic or causal relationship.

Maxillary lateral wall thickness, a structure frequently involved in surgically assisted rapid maxillary expansion (SARME), also exhibits marked interindividual variability and has been identified as an important contributor to skeletal resistance [13, 14, 15, 16, 17, 18, 19, 20, 21]. Although not assessed in the present study, this parameter represents a relevant avenue for future investigations, particularly when combined with facial growth pattern and MPS morphology.

The relatively low explanatory power of the regression model (Cox & Snell R^2^ = 0.0451) is consistent with the multifactorial nature of craniofacial growth and sutural maturation, which are influenced by genetic, anatomical, functional and environmental factors [5, 12, 16]. Such values are common in biological research and do not invalidate statistically significant associations. Importantly, the ordered thresholds between maturation stages demonstrated biological coherence, supporting the interpretation of the Angelieri classification as an ordinal diagnostic framework rather than a precise predictive scale. The borderline significance observed for the transition between stages D and E likely reflects the biological variability inherent to late‐stage sutural fusion in adults, as previously reported in tomographic and histological studies [5, 16].

CBCT remains the reference standard for evaluating MPS maturation due to its ability to provide detailed three‐dimensional visualisation of sutural morphology and adjacent skeletal structures [3, 4]. Nevertheless, the present findings suggest that facial growth pattern may serve as a complementary, low‐cost and radiation‐free diagnostic parameter. Because facial pattern classification is routinely derived from standard cephalometric analysis, it may help identify cases in which CBCT evaluation is particularly justified.

This study has limitations that must be acknowledged. The sample consisted of Brazilian adults with heterogeneous ethnic backgrounds, and population‐related differences in craniofacial maturation patterns have been reported [18, 19, 20, 21, 22, 23, 24, 25]. In addition, the retrospective cross‐sectional design precludes assessment of temporal changes in sutural maturation. Although 132 scans met the inclusion criteria, only 120 were used for stratified allocation, and the remaining cases were not employed for secondary validation. Future longitudinal and multicentre studies are warranted to explore how facial growth patterns interact with sutural maturation over time and across diverse populations.

In summary, facial growth pattern is associated with MPS maturation stage in adults, reinforcing its value as an adjunctive diagnostic parameter. Similar to other anatomical indicators used in craniofacial assessment, facial type provides clinically meaningful information regarding skeletal morphology and sutural status, but should be interpreted as a complementary tool rather than a substitute for CBCT‐based evaluation. These findings support a more individualised and anatomy‐oriented approach to the diagnosis of transverse maxillary deficiency in adult patients.

## 5 Conclusions

Facial growth pattern is associated with MPS maturation stage in adults, with dolichofacial individuals more frequently presenting earlier stages of sutural maturation, whereas age and sex showed no significant association. These findings indicate that vertical skeletal morphology reflects anatomical variability related to sutural development and may function as an adjunctive diagnostic parameter in orthodontic assessment. However, facial pattern analysis should not replace CBCT, and the results must be interpreted cautiously, given the cross‐sectional design and specific population studied, reinforcing the need for longitudinal and multicentre investigations.

## Author Contributions

R.S.O. made substantial contributions to the conception and design of the study, as well as to data acquisition, analysis and interpretation. R.S.O., E.N.S., F.B.S., F.K.P., and N.M.R.A. were involved in drafting the manuscript and revising it critically for important intellectual content. R.S.O., N.M.R.A, F.B.S., F.K.P., and E.N.S. approved the final version to be published. All authors participated sufficiently in the work to take public responsibility for appropriate portions of the content.

## Funding

This study was supported by CAPES‐BRAZIL (Grant No. 88887.808604/2023–00).

## Ethics Statement

This retrospective observational study was approved by the Institutional Research Ethics Committee (Protocol No. 01617318.0.0000.5374) and was conducted in accordance with the principles of the Declaration of Helsinki.

## Consent

Due to the retrospective design of this study and the use of anonymised imaging data, the requirement for informed consent was waived by the Institutional Research Ethics Committee.

## Conflicts of Interest

The authors declare no conflicts of interest.

## Supporting information


**Data S1:** Determination of facial growth pattern (A—brachyfacial, B—mesofacial and C—dolichofacial) using Ricketts' VERT index from lateral Cephalometric tracings reconstructed with Dolphin Imaging software.

## Data Availability

The data that support the findings of this study are available from the corresponding author upon reasonable request.
