# Supplementary material for: Facial Growth Patterns and Midpalatal Suture Maturation in Adults: A Retrospective CBCT‐Based Observational Study
Source: Orthod Craniofac Res. 2026 Mar 27;29(4):708–16. doi: 10.1111/ocr.70118 (PMC13485212; doi:10.1111/ocr.70118)
Supplement: Supplementary file 1 [file OCR-29-708-s001.pdf]

Supplementary Material

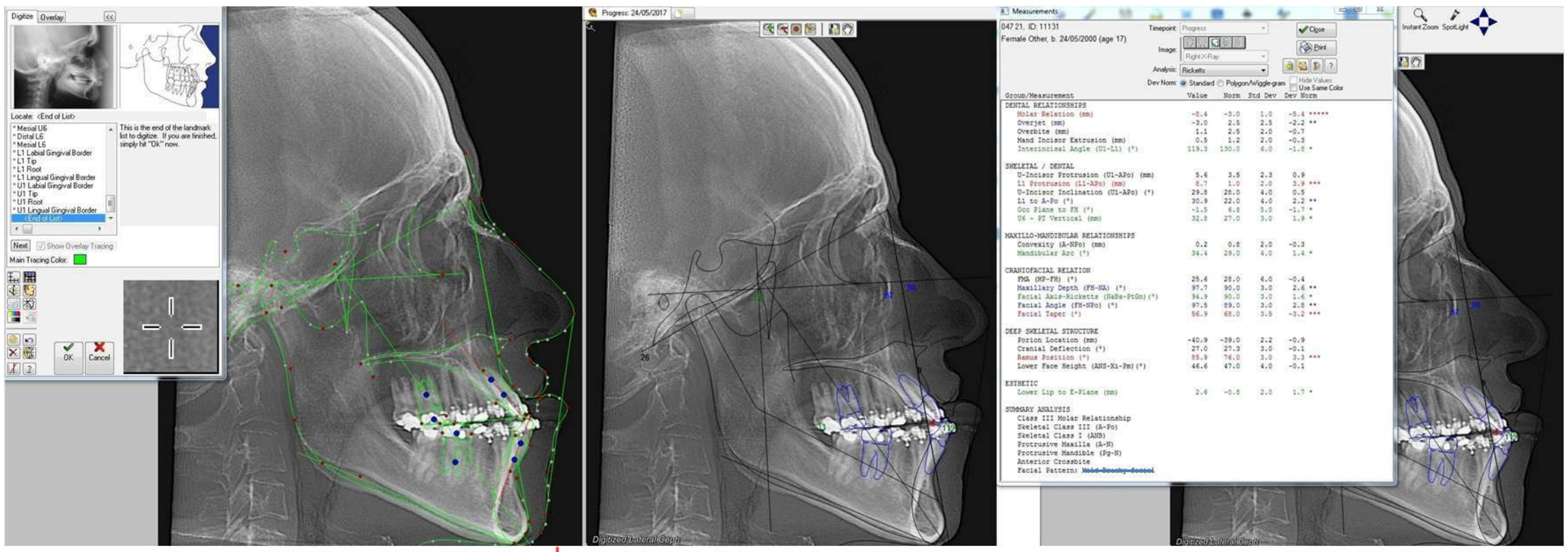

**DENTAL RELATIONSHIPS**			
Molar Relation (mm)	0,2	-3,0	1,0
Overjet (mm)	0,4	2,5	2,5
Overbite (mm)	-2,9	2,5	2,0
Mand Incisor Extrusion (	-1,5	1,2	2,0
Interincisal Angle (U1-L1	114,5	130,0	6,0
**SKELETAL / DENTAL**			
U-Incisor Protrusion (U1-	4,9	3,5	2,3
L1 Protrusion (L1-APo) (	4,2	1,0	2,3
U-Incisor Inclination (U1	30,5	28,0	4,0
L1 to A-Po (°)	35,1	22,0	4,0
Occ Plane to FH (°)	1,9	4,7	5,0
U6 - PT Vertical (mm)	20,8	21,0	3,0
**MAXILLO-MANDIBULAR RELATIONSHIPS**			
Convexity (A-NPo) (mm)	-0,8	0,1	2,0
Mandibular Arc (°)	35,9	31,7	4,0
**CRANIOFACIAL RELATION**			
FMA (MP-FH) (°)	17,6	22,9	4,5
Maxillary Depth (FH-NA)	92,7	90,0	3,0
Facial Axis-Ricketts (NaB	97,7	90,0	3,5
Facial Angle (FH-NPo) (°	93,6	89,6	3,0
Facial Taper (°)	68,8	68,0	3,5
**DEEP SKELETAL STRUCTURE**			
Porion Location (mm)	-36,5	-38,6	2,2
Cranial Deflection (°)	24,7	27,3	3,0
Ramus Position (°)	78,6	76,0	3,0
Lower Face Height (ANS-	35,3	45,0	4,0
**ESTHETIC**			
Lower Lip to E-Plane (mr	-1,5	-2,0	2,0

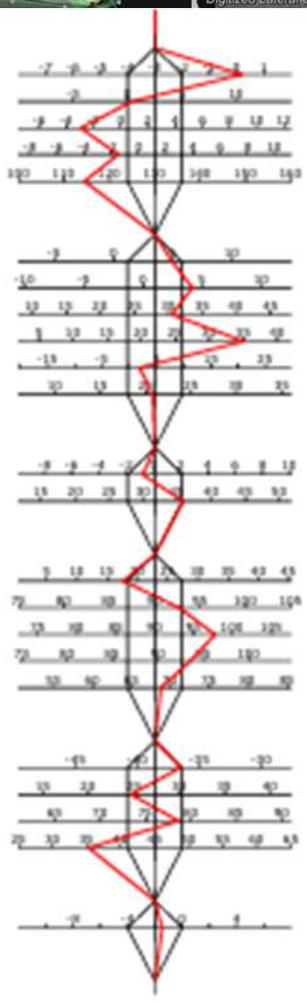

SUMMARY ANALYSIS

- Class II Molar Relationship
- Skeletal Class III (A-Po)
- Skeletal Class I (ANB)
- Low Mandibular Plane Angle
- Protrusive Mandible (Pg-N)
- Open Bite
- Facial Pattern: Brachy-facial

A

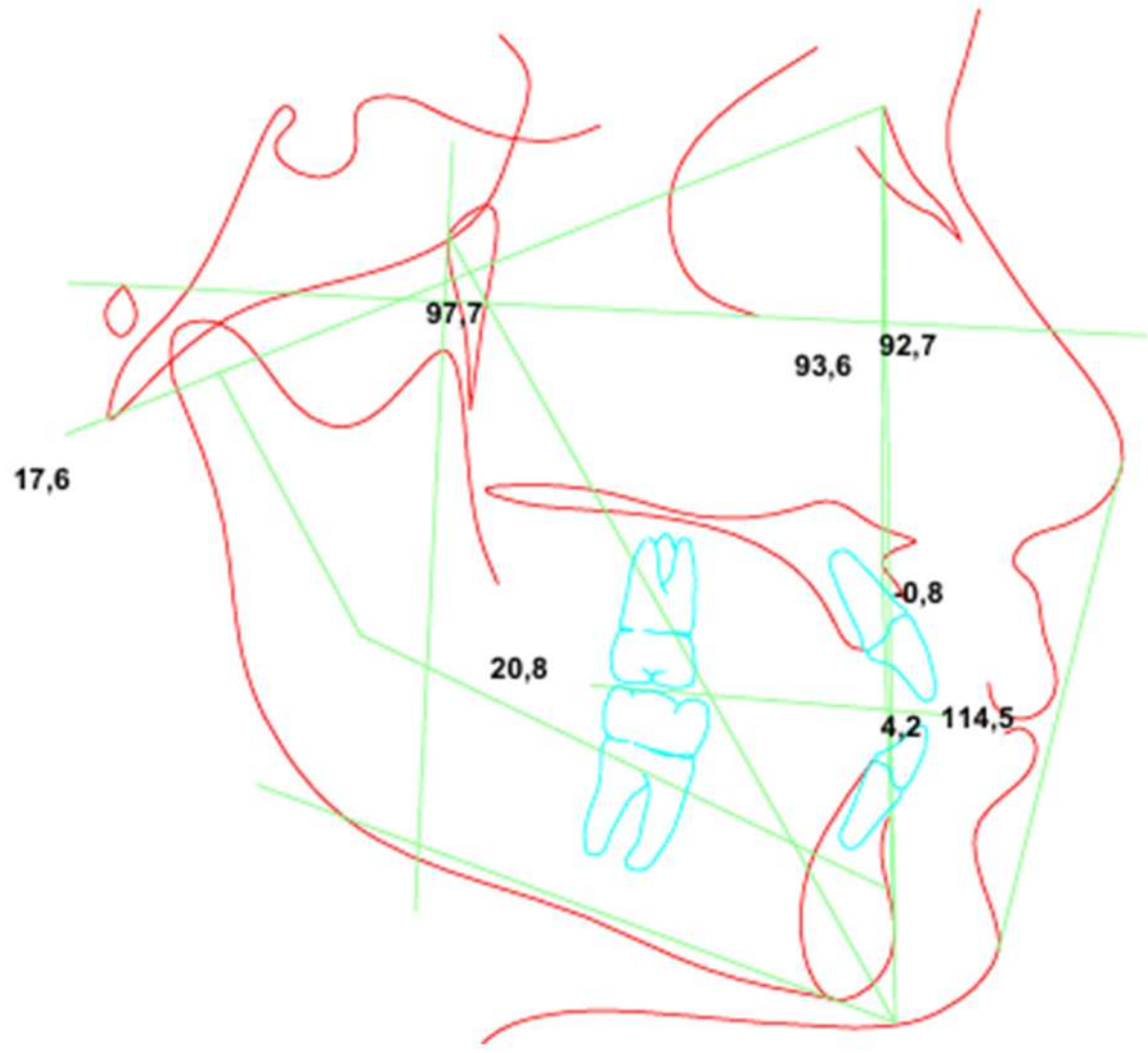

**DENTAL RELATIONSHIPS**			
Molar Relation (mm)	-0,3	-3,0	1,0
Overjet (mm)	7,0	2,5	2,5
Overbite (mm)	0,9	2,5	2,0
Mand Incisor Extrusion (	0,4	1,2	2,0
Interincisal Angle (U1-L1	116,6	130,0	6,0
**SKELETAL / DENTAL**			
U-Incisor Protrusion (U1-	9,5	3,5	2,3
L1 Protrusion (L1-APo) (	2,5	1,0	2,3
U-Incisor Inclination (U1	36,6	28,0	4,0
L1 to A-Po (°)	26,7	22,0	4,0
Occ Plane to FH (°)	4,1	5,3	5,0
U6 - PT Vertical (mm)	17,8	20,1	3,0
**MAXILLO-MANDIBULAR RELATIONSHIPS**			
Convexity (A-NPo) (mm)	2,6	0,3	2,0
Mandibular Arc (°)	33,9	31,2	4,0
**CRANIOFACIAL RELATION**			
FMA (MP-FH) (°)	26,2	23,2	4,5
Maxillary Depth (FH-NA)	91,4	90,0	3,0
Facial Axis-Ricketts (NaB	89,7	90,0	3,5
Facial Angle (FH-NPo) (°	88,7	89,3	3,0
Facial Taper (°)	65,1	68,0	3,5
**DEEP SKELETAL STRUCTURE**			
Porion Location (mm)	-37,6	-38,6	2,2
Cranial Deflection (°)	27,8	27,3	3,0
Ramus Position (°)	77,4	76,0	3,0
Lower Face Height (ANS-	43,3	45,0	4,0
**ESTHETIC**			
Lower Lip to E-Plane (mr	-1,2	-2,0	2,0

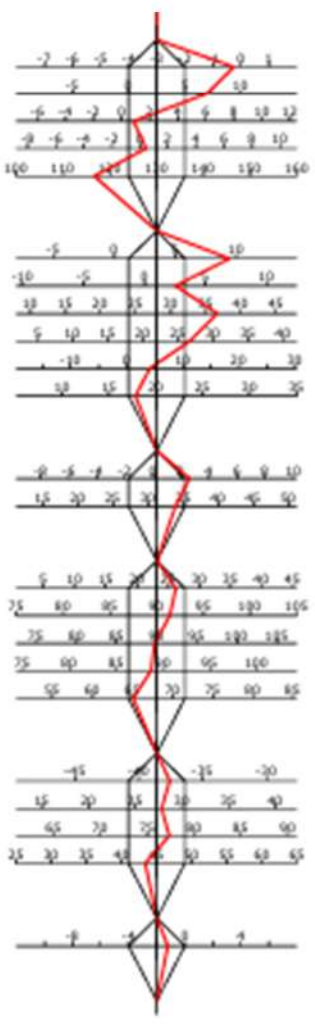

SUMMARY ANALYSIS

- Class II Molar Relationship
- Skeletal Class II (A-Po)
- Skeletal Class II (ANB)
- Excessive Overjet
- Facial Pattern: Meso-facial

B

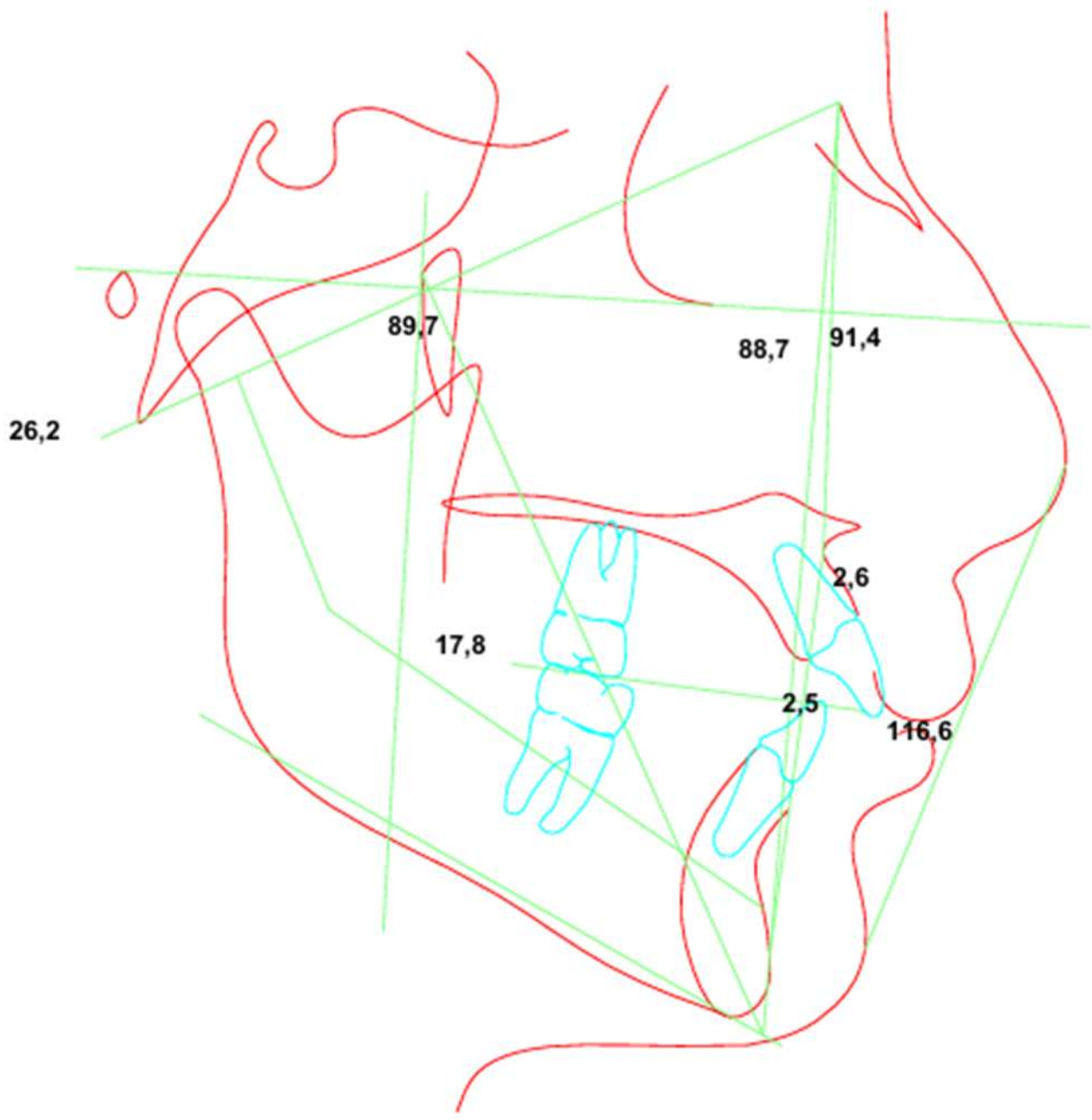

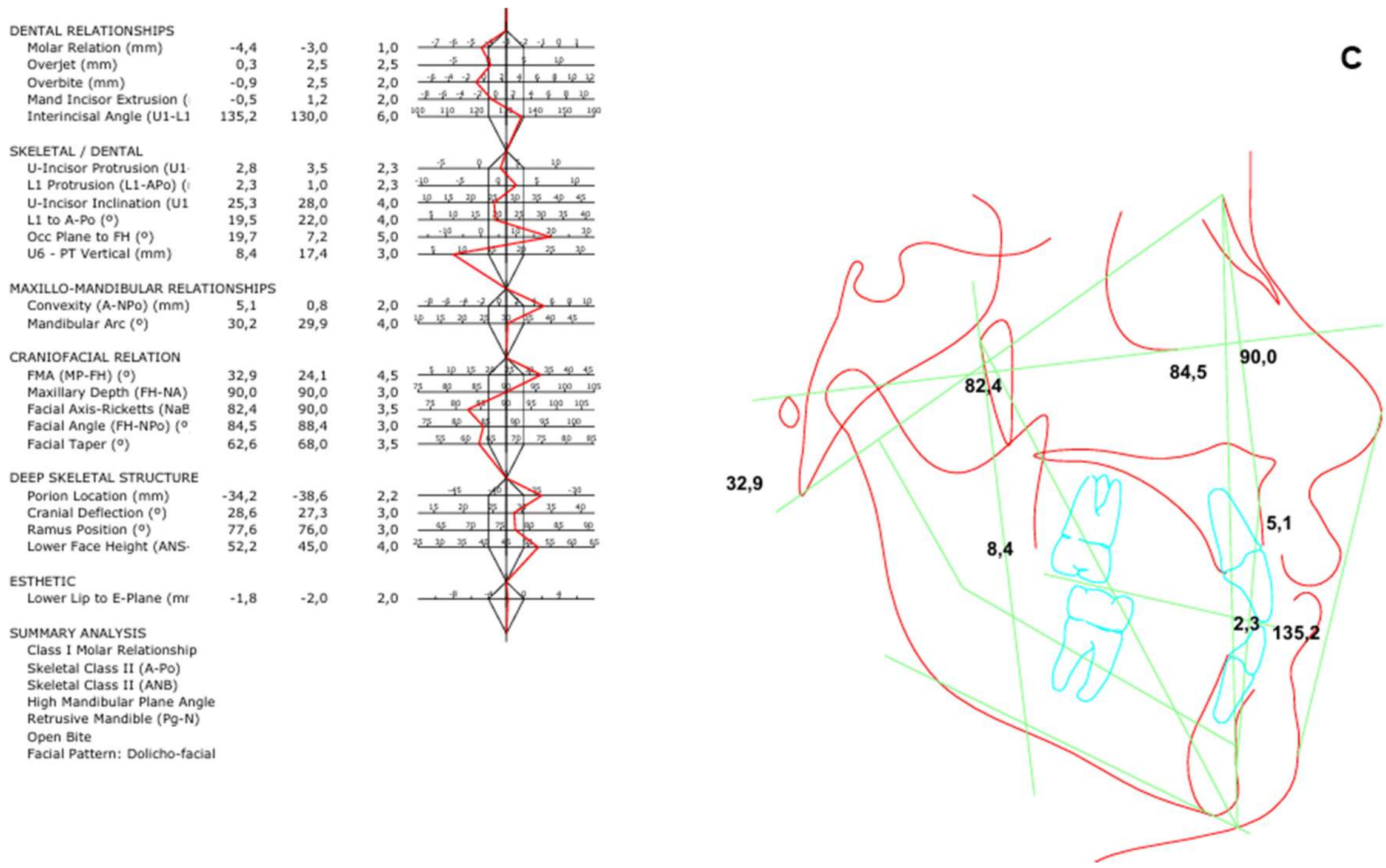

Determination of facial growth pattern (A- brachyfacial, B - mesofacial, C- dolichofacial) using Ricketts' VERT index from lateral Cefalometria tracings reconstructed with Dolphin® Imaging software.
